# Association between the non-high-density lipoprotein cholesterol to high-density lipoprotein cholesterol ratio and post-stroke depression in patients with acute ischemic stroke

**DOI:** 10.3389/fpsyt.2026.1882234

**Published:** 2026-10-12

**Authors:** Yu Yun, Jie Liu, Yaqiang Li

**Affiliations:** Department of Neurology, People’s Hospital of Lixin County, Bozhou, Anhui, China

**Keywords:** biomarker, inflammation, non-high-density lipoprotein cholesterol to high-density lipoprotein cholesterol ratio (NHHR), post-stroke depression (PSD), stroke

## Abstract

**Objectives:**

Post-stroke depression (PSD) is a frequent and debilitating complication of acute ischemic stroke (AIS), yet its underlying biochemical predictors remain insufficiently characterized. The non-high-density lipoprotein cholesterol to high-density lipoprotein cholesterol ratio (NHHR) has recently emerged as a robust composite metric for assessing the homeostatic imbalance of lipid metabolism and systemic inflammation. This study aimed to evaluate the longitudinal association between admission NHHR levels and the risk of developing PSD at three months post-stroke in patients with AIS.

**Methods:**

We consecutively enrolled 408 patients with AIS from the Department of Neurology at Lixin County People’s Hospital between February 2023 and January 2026. Admission NHHR was calculated from fasting lipid panels. Depressive symptoms were evaluated at a 3-month follow-up using the 17-item Hamilton Depression Rating Scale (HAMD-17), with PSD diagnosis confirmed via DSM-V criteria. Participants were stratified into tertiles based on admission NHHR (Q1 ≤7.68; Q2 7.69–10.50; Q3 ≥10.52). Multivariate logistic regression and restricted cubic splines (RCS) were employed to analyze the association between NHHR and PSD risk.

**Results:**

Among the 408 participants, 93 (22.79%) were diagnosed with PSD. Patients in the PSD group exhibited significantly higher admission NHHR levels compared to the non-PSD group (*P* < 0.001). The prevalence of PSD increased progressively across NHHR tertiles: 10.75% in Q1, 25.81% in Q2, and 63.44% in Q3. After adjusting for age, sex, vascular risk factors, stroke severity (NIHSS), and functional impairment (mRS), the highest NHHR tertile remained a potent independent predictor of PSD (OR 3.240, 95% CI 1.850–5.680, *P* < 0.001). The admission NHHR level exhibited a robust predictive capacity for PSD, with an AUC of 0.738 (95% CI: 0.681–0.790, *P* < 0.001). RCS analysis further revealed a statistically significant non-linear relationship (*P* for non-linearity < 0.001), identifying a vulnerability window between NHHR values of 10 and 15 where the risk of depression escalated most sharply. This association persisted in various subgroup analyses.

**Conclusions:**

Elevated admission NHHR is independently and non-linearly associated with an increased risk of PSD at three months in AIS patients. As an easily accessible biomarker of lipid-driven systemic inflammation, NHHR may serve as a valuable prognostic tool for early risk stratification and targeted psychological intervention in clinical practice.

## 1 Introduction

Acute ischemic stroke (AIS) remains a leading cause of long-term disability and mortality worldwide, imposing a significant socioeconomic burden (1, 2). Beyond physical impairments, the neuropsychiatric sequelae of stroke—most notably Post-Stroke Depression (PSD)—have emerged as critical determinants of clinical outcomes (3, 4). PSD affects approximately one-third of stroke survivors, significantly hindering functional recovery, increasing the risk of recurrent cerebrovascular events, and escalating all-cause mortality (5, 6). Despite its prevalence, the complex pathophysiology of PSD, which likely involves an interplay of neuroinflammation, oxidative stress, and neuroendocrine dysregulation, remains incompletely understood (7–9). Consequently, identifying reliable and easily accessible biomarkers for the early stratification of PSD risk is of paramount importance in contemporary vascular neurology.

The role of lipid metabolism in the pathogenesis of both cerebrovascular diseases and affective disorders has garnered increasing attention (10–12). Traditionally, individual lipid parameters such as Low-Density Lipoprotein Cholesterol (LDL-C) and High-Density Lipoprotein Cholesterol (HDL-C) have been studied as cardiovascular risk factors (13, 14). However, these conventional markers often fail to capture the comprehensive pro-atherogenic lipid profile and the underlying systemic inflammation. In recent years, composite lipid indices have been proposed as more robust predictors of metabolic and vascular health. Non-high-density lipoprotein cholesterol (non-HDL-C) encompasses the entire spectrum of pro-atherogenic lipoproteins, effectively serving as a comprehensive surrogate for cholesterol-mediated vascular injury (15). By integrating this atherogenic burden with the protective capacity of HDL, the non-HDL-C to HDL-C ratio (NHHR) emerges as a refined composite metric that captures the homeostatic imbalance of lipid metabolism more accurately than traditional parameters. Recent evidence has underscored the potent predictive utility of NHHR across a broad landscape of pathological conditions, extending beyond cerebrovascular and cardiovascular events to include systemic inflammation and depression (16–19).

To date, few studies have specifically examined the longitudinal association between NHHR measured at the acute stage of ischemic stroke and the subsequent development of PSD. Given the ease of calculating NHHR from routine fasting lipid panels, its validation as a prognostic tool could significantly enhance clinical decision-making. Identifying high-risk individuals early in the post-stroke phase would allow for targeted psychological interventions and pharmacological prophylaxis, potentially improving the quality of life and functional prognosis for AIS patients. Therefore, the present study aims to evaluate the association between admission NHHR levels and the risk of PSD at three months post-stroke in a large, well-characterized cohort of patients with AIS.

## 2 Materials and methods

### 2.1 Subjects

Between February 2023 and January 2026, patients with AIS were consecutively enrolled from the Department of Neurology at Lixin County People’s Hospital. Initially, 487 participants were screened for eligibility. Patients were excluded based on the following pre-specified criteria (1): a documented history of depression or other psychiatric disorders (n=5) (2); severe dysarthria or aphasia hindering assessment (n=8) (3); severe hepatic or renal dysfunction, or underlying malignancy (n=10) (4); active infection within two weeks prior to stroke onset (defined by clinical symptoms, such as an axillary temperature ≥ 37.3 °C or respiratory/urinary symptoms, combined with laboratory confirmation including Creactive protein > 10 mg/L, procalcitonin > 0.05 ng/mL, or white blood cell count > 10.0 × 10³/μL) (n=7) (5); diagnosis of transient ischemic attack (TIA, n=18) (6); insufficient laboratory data regarding serum lipid levels (n=12); and (7) loss to follow-up at the 3-month mark (n=19). Consequently, a total of 408 patients were ultimately included in the final analysis (**Figure 1**). This study received formal approval from the institutional ethics committee of Lixin County People’s Hospital (Ethics Approval No. LXXRMYY-2023KY04) and was conducted in strict accordance with the Declaration of Helsinki. Written informed consent was obtained from all participants or their legally authorized representatives prior to enrollment.

**Figure 1:**
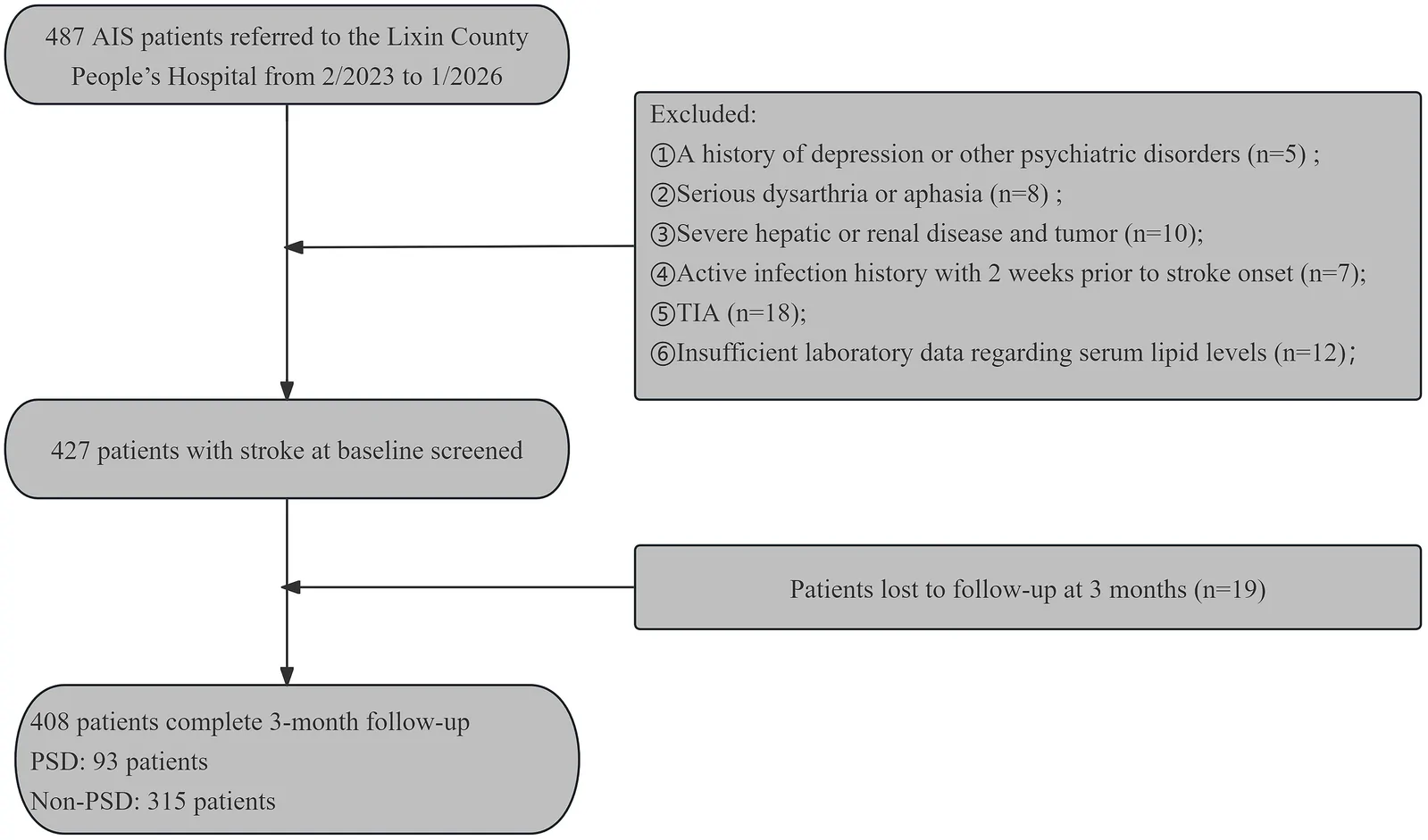
Flowchart of participant selection.

### 2.2 Data collection

Comprehensive baseline data were systematically retrieved from electronic medical records. Demographic variables encompassed age, sex, marital status, and educational attainment. Vascular risk profiles were documented, including hypertension, diabetes mellitus, coronary heart disease, atrial fibrillation, smoking status, alcohol consumption, and history of prior stroke. Neuroimaging findings, derived from admission computed tomography or magnetic resonance imaging, were reviewed to confirm the diagnosis and infarct location. Stroke severity was quantified by certified neurologists upon admission using the National Institutes of Health Stroke Scale (NIHSS) (20). Clinical outcomes and cognitive status were evaluated during a scheduled 3-month follow-up via the Modified Rankin Scale (mRS) and the Barthel Index (BI) for functional independence, alongside the Mini-Mental State Examination (MMSE) for cognitive screening. All assessments were performed by trained clinicians blinded to the laboratory results to ensure objectivity (21–23).

### 2.3 Blood collection and laboratory test

Venous blood samples were collected consecutively from all AIS participants within 24 hours of admission, following a standardized fasting period of at least 8 hours. To minimize bias, laboratory personnel were blinded to the clinical and neuroimaging data. Full blood counts, including white blood cell (WBC), neutrophil (N), lymphocyte (L), and monocyte counts, were determined using the Sysmex XE-2100 Hematology Automated Analyzer (Sysmex, Kobe, Japan). Biochemical parameters, including fasting plasma glucose (FPG), total cholesterol (TC), triglycerides (TG), high-density lipoprotein cholesterol (HDL-C), low-density lipoprotein cholesterol (LDL-C), homocysteine (Hcy), and apolipoproteins (ApoA and ApoB), were quantified via the AU5811 automatic biochemical analyzer (Beckman Coulter, USA). Non-HDL-C was derived by subtracting HDL-C from TC. The non-HDL-C to HDL-C ratio (NHHR) and the neutrophil-to-lymphocyte ratio (NLR) were subsequently calculated as the quotients of non-HDL-C/HDL-C and N/L, respectively. All laboratory protocols strictly adhered to standardized manufacturer instructions and quality control procedures.

### 2.4 Psychological measurement

Depressive symptoms were systematically evaluated during the 3-month follow-up via face-to-face or standardized telephone interviews using the 17-item Hamilton Depression Rating Scale (HAMD-17) (24). To ensure diagnostic gold-standard accuracy, patients scoring >7 on the HAMD-17 were further assessed to confirm a diagnosis of PSD, concordant with the Diagnostic and Statistical Manual of Mental Disorders, 5th Edition (DSM-V) criteria. All clinical psychological evaluations were independently administered by two experienced researchers who had undergone pre-study standardized training to ensure consistency. The inter-rater reliability was formally assessed using Cohen’s kappa coefficient, which yielded an excellent agreement value of κ = 0.86. To maintain objectivity and minimize assessment bias, these investigators remained strictly blinded to the patients’ baseline laboratory findings and neuroimaging data throughout the study period.

### 2.5 Statistical analysis

The Kolmogorov–Smirnov test was initially applied to assess the distributional normality of continuous variables. Normally distributed data are presented as mean ± standard deviation (SD) and were compared using Student’s t-test or one-way analysis of variance (ANOVA). Non-normally distributed variables are expressed as median with interquartile range (IQR); differences between two or three groups were evaluated via the Mann–Whitney U test or Kruskal–Wallis test, respectively. Categorical variables are reported as frequencies (percentages) and were analyzed using the chi-square test. Patients were stratified into tertiles based on admission NHHR levels (Q1 ≤ 7.68; Q2 7.69–10.50; Q3 ≥ 10.52). The independent association between NHHR and PSD was determined through stepwise multivariate logistic regression models: Model 1 adjusted for age and sex; Model 2 further included vascular risk factors; and Model 3 adjusted for all univariate-significant covariates (*P* < 0.05), including HDL-C, basal ganglia lesions, baseline NIHSS score, and mRS score. To explore potential non-linear relationships and the dose-response pattern, restricted cubic splines (RCS) with four knots (at the 5th, 35th, 65th, and 95th percentiles) were constructed, adjusting for the same covariates as Model 3. The Receiver Operating Characteristic (ROC) curve analysis was performed. The Area Under the Curve (AUC), optimal cut-off value (determined by the maximum Youden index), sensitivity, and specificity with their corresponding 95% confidence intervals (CIs) were calculated. Furthermore, to explore population heterogeneity and potential effect modifications, stratified subgroup analyses were conducted across clinical and demographic factors. Interactive terms were tested using logistic regression models to obtain *P* for interaction. All analyses were performed using R (version 4.4.2) and the Storm Statistical Platform (http://www.medsta.cn/software). A two-tailed *P* < 0.05 was considered statistically significant.

## 3 Results

### 3.1 Baseline characteristics of patients in PSD group and non-PSD group

A total of 487 consecutive patients diagnosed with AIS were initially recruited from one clinical center between February 2023 and January 2026. Following application of the predefined exclusion criteria—including prior psychiatric disorders (n=5), severe communication barriers (n=8), significant comorbidities (n=10), active infection (n=7), TIA (n=18), insufficient lipid data (n=12), and loss to follow-up (n=19)—79 patients were excluded. Consequently, 408 patients (177 female; mean age 66.16 ± 8.43 years) were enrolled in the final analysis.

**Table 1** summarizes the demographic, clinical, and laboratory characteristics of the study population. Among the 408 participants, 93 (22.79%) were diagnosed with PSD at the 3-month follow-up. While demographics, vascular risk factors, and lesion locations were comparable between groups, patients with PSD exhibited significantly higher baseline NIHSS scores (*P* < 0.001), mRS scores (*P* < 0.001) and basal ganglia (*P* = 0.001), alongside lower BI scores (*P* < 0.001), indicating greater neurological impairment and functional disability. Notably, PSD was associated with significantly elevated levels of WBC, neutrophils, NLR, Hcy, and NHHR (all *P* < 0.05), whereas HDL-C levels were significantly lower (*P* < 0.05) compared to the non-PSD group.

**Table 1:** Clinical and demographic characteristics of patients with PSD and non-PSD.

Variables	Total (n=408)	Depression after stroke		*P* value
		Depression (n=93)	Non-depression (n=315)	
Demographic characteristics				
Gender, female, n (%)	177 (43.38)	46 (49.46)	131 (41.59)	0.617
Age, years, mean ± SD	66.16 ± 8.43	65.77 ± 8.98	66.27 ± 8.27	0.421
Education years, median (IQR)	5 (3–8)	5 (3–8)	5 (3–8)	0.712
Married, n(%)	385 (94.36)	88 (94.62)	297 (94.29)	0.899
Vascular risk factors (%)				
Hypertension	263 (64.46)	62 (66.67)	201 (63.81)	0.702
Diabetes mellitus	131 (32.11)	31 (33.33)	100 (31.75)	0.872
Coronary heart disease	51 (12.50)	12 (12.90)	39 (12.38)	0.891
Atrial fibrillation	13 (3.19)	2 (2.15)	11 (3.49)	0.741
current smoking	109 (26.72)	27 (29.03)	82 (26.03)	0.659
Alcohol consumption	130 (31.86)	29 (31.18)	101 (32.06)	0.973
Prior stroke	77 (18.87)	19 (20.43)	58 (18.41)	0.775
Laboratory findings (IQR)				
WBC, ×10^9^/L, median (IQR)	6.57 (4.85-8.93)	7.74 (5.13-9.61)	6.40 (4.69-8.72)	0.023
L, ×10^9^/L, median (IQR)	1.13 (0.81-1.55)	1.14 (0.84-1.44)	1.13 (0.79-1.61)	0.891
N, ×10^9^/L, median (IQR)	4.66 (3.22-6.38)	5.43 (3.67-7.46)	4.86 (3.02-6.08)	0.004
NLR, median (IQR)	4.04 (2.73-6.53)	5.09 (3.42-6.96)	4.81 (3.62-5.85)	0.041
Glucose, mmol/L, median (IQR)	5.30 (4.70-6.80)	5.40 (4.70-7.40)	5.30 (4.70-6.70)	0.519
TG, mmol/L, median (IQR)	1.26 (1.00-1.63)	1.23 (0.99-1.52)	1.30 (1.00-1.65)	0.297
TC, mmol/L, median (IQR)	5.29 (4.89-5.83)	5.20 (4.88-5.71)	5.30 (4.90-5.86)	0.492
HDL-C, mmol/L, median (IQR)	1.03 (0.96-1.31)	0.98 (0.93-1.29)	1.16 (0.98-1.63)	0.012
LDL-C, mmol/L, median (IQR)	1.24 (0.89-1.62)	1.12 (0.81-1.53)	1.26 (0.96-1.63)	0.072
ApoA, g/L, median (IQR)	1.26 (1.10-1.43)	1.26 (1.12-1.45)	1.26 (1.09-1.43)	0.434
ApoB, g/L, median (IQR)	0.86 (0.70-1.02)	0.86 (0.70-1.05)	0.86 (0.70-1.00)	0.471
Hcy, µmol/L, median (IQR)	12.72 (10.25-16.21)	13.83 (11.37-16.80)	12.32 (10.00-15.47)	0.008
Non-HDL-C, mmol/L, median (IQR)	5.05 (4.70-5.51)	5.06 (4.73-5.49)	5.05 (4.70-5.52)	0.863
NHHR	9.30 (7.2-11.62)	11.59 (9.87-16.00)	8.59 (6.78-11.50)	< 0.001
Lesion location, n(%)				
Frontal lobe	78 (19.12)	20 (21.51)	58 (18.41)	0.606
Parietal lobe	77 (18.87)	20 (21.51)	57 (18.10)	0.557
Temporal lobe	88 (21.57)	21 (22.58)	67 (21.27)	0.899
Occipital lobe	60 (14.71)	11 (11.83)	49 (15.56)	0.468
Basal ganglia	110 (26.96)	38 (40.86)	72 (22.86)	0.001
Brainstem	76 (18.63)	18 (19.35)	58 (18.41)	0.957
Cerebellum	25 (6.13)	8 (8.60)	17 (5.40)	0.375
Neuropsychological function				
NIHSS score, median (IQR)	6 (4-9)	9 (7-11)	6 (4-8)	<0.001
MMSE score, median (IQR)	22.00 (17.00-26.00)	22.00 (17.00-26.00)	22.00 (17.00-26.00)	0.893
BI score, median (IQR)	75.00 (50.00-90.00)	60.00 (40.00-75.00)	80.00 (60.00-95.00)	<0.001
mRS score, median (IQR)	3.00 (2.00-4.00)	3.00 (3.00-4.00)	2.00 (2.00-3.00)	<0.001
HAMD score, median (IQR)	5.00 (2.00-6.25)	11.00 (9.00-17.00)	3.00 (2.00-5.50)	<0.001

WBC, White blood cell; N, neutrophil; L, lymphocyte; NLR, neutrophil-to-lymphocyte ratio; TG, total triglyceride; TC, total cholesterol; LDL-C, low density lipoprotein cholesterol; HDL-C, high density lipoprotein cholesterol; ApoA, apolipoprotein A; ApoB, apolipoprotein B; Hcy, homocysteine; NHHR, Non-High-Density Lipoprotein Cholesterol to High-Density Lipoprotein Cholesterol Ratio; BI, Barthel Index; mRS, modified Rankin Scale; NIHSS, National Institute of Health Stroke Scale; MMSE, Mini-Mental State Examination; IQR, inter-quartile range.

### 3.2 Baseline characteristics of all patients in NHHR tertiles

Based on the data in **Table 2**, patients were categorized into three groups according to their NHHR levels: Q1 (≤7.68), Q2 (7.69–10.50), and Q3 (≥10.52). The analysis reveals that higher NHHR levels are significantly associated with increased stroke severity and worse clinical outcomes. Patients in the Q3 group demonstrated significantly higher NIHSS scores (*P* = 0.001) and mRS scores (*P* = 0.015), indicating more severe neurological deficits and higher disability levels. Additionally, higher NHHR was linked to increased psychological distress, as evidenced by elevated HAMD scores (*P* < 0.001). Regarding lesion location, Q3 patients showed a significantly higher prevalence of basal ganglia involvement (*P* = 0.002). Conversely, higher NHHR correlated with lower HDL-C levels (*P* = 0.011). Demographic factors and vascular risks remained consistent across all groups. A significant disparity in PSD incidence was observed across tertiles (χ2 = 27.639 for Q1; χ2 = 49.135 for Q3; *P* < 0.001). The prevalence of depression increased progressively with NHHR levels: 10.75% in Q1, 25.81% in Q2, and 63.44% in Q3 (**Table 3**).

**Table 2:** Baseline characteristics of patients with AIS according to NHHR tertiles.

Variables	NHHR			*P* value
	Q1 (≤ 7.68, n=136)	Q2 (7.69-10.50, n=136)	Q3 (≥10.52, n=136)	
Demographic characteristics				
Gender, female, n (%)	62 (45.59)	65 (47.79)	68 (50.00)	0.767
Age, years, median (IQR)	68.00 (63.00-71.00)	68.00 (61.00-71.00)	68.00 (59.75-73.00)	0.913
Education years, median (IQR)	5.00 (3.00-7.00)	5.00 (3.00-7.25)	5.50 (3.00-8.00)	0.291
Married, n(%)	128 (94.12)	127 (93.38)	130 (95.59)	0.724
Vascular risk factors (%)				
Hypertension	87 (63.97)	86 (63.24)	90 (66.18)	0.870
Diabetes mellitus	39 (28.68)	46 (33.82)	46 (33.82)	0.576
Coronary heart disease	14 (10.29)	14 (10.29)	21 (15.44)	0.321
Atrial fibrillation	3 (2.21)	8 (5.88)	2 (1.47)	0.162
current smoking	34 (25.00)	38 (27.94)	37 (27.21)	0.850
Alcohol consumption	39 (28.68)	42 (30.88)	49 (36.03)	0.410
Prior stroke	24 (17.65)	23 (16.91)	26 (19.12)	0.890
Laboratory findings (IQR)				
WBC, ×10^9^/L, median (IQR)	6.25 (4.49-8.63)	6.49 (5.03-8.73)	7.08 (5.15-9.32)	0.072
L, ×10^9^/L, median (IQR)	1.18 (0.86-1.63)	1.13 (0.74-1.53)	1.10 (0.80-1.52)	0.352
N, ×10^9^/L, median (IQR)	4.47 (2.90-6.10)	4.57 (3.20-6.14)	4.63 (3.57-6.36)	0.057
NLR, median (IQR)	3.60 (2.33-4.99)	3.98 (2.69-6.27)	4.09 (3.31-6.98)	0.051
Glucose, mmol/L, median (IQR)	5.20 (4.60-6.62)	5.30 (4.70-6.90)	5.40 (4.77-6.90)	0.390
TG, mmol/L, median (IQR)	1.34 (1.01-1.66)	1.28 (1.02-1.61)	1.18 (0.99-1.49)	0.067
TC, mmol/L, median (IQR)	5.30 (4.88-5.81)	5.46 (5.02-6.07)	5.34 (4.88-5.60)	0.054
HDL-C, mmol/L, median (IQR)	1.45 (1.12-2.08)	1.04 (0.99-1.70)	0.92 (0.87-1.38)	0.011
LDL-C, mmol/L, median (IQR)	1.29 (0.89-1.62)	1.32 (0.96-1.66)	1.22 (0.88-1.38)	0.051
ApoA, g/L, median (IQR)	1.25 (1.10-1.42)	1.27 (1.10-1.45)	1.26 (1.11-1.42)	0.610
ApoB, g/L, median (IQR)	0.84 (0.69-1.00)	0.85 (0.69-1.02)	0.89 (0.72-1.03)	0.557
Hcy, µmol/L, median (IQR)	12.53 (10.18-16.49)	12.47 (10.01-16.22)	12.92 (10.63-15.31)	0.849
Non-HDL-C, mmol/L, median (IQR)	4.98 (4.50-5.54)	5.22 (4.72-5.88)	5.19 (4.70-5.48)	0.057
NHHR, median (IQR)	6.41 (5.49-7.22)	9.30 (8.57-10.00)	13.26 (11.65-16.03)	< 0.001
Lesion location, n(%)				
Frontal lobe	33 (24.26)	23 (16.91)	22 (16.18)	0.172
Parietal lobe	20 (14.71)	25 (18.38)	32 (23.53)	0.175
Temporal lobe	33 (24.26)	33 (24.26)	22 (16.18)	0.173
Occipital lobe	19 (13.97)	24 (17.65)	17 (12.50)	0.467
Basal ganglia	24 (17.65)	36 (26.47)	50 (36.76)	0.002
Brainstem	22 (16.18)	26 (19.12)	28 (20.59)	0.636
Cerebellum	6 (4.41)	8 (5.88)	11 (8.09)	0.445
Neuropsychological function				
NIHSS score, median (IQR)	6.00 (4.00-8.00)	6.00 (4.00-8.00)	7.00 (5.00-10.00)	0.001
MMSE score, median (IQR)	22.00 (17.75-26.00)	20.00 (16.00-26.00)	24.00 (18.00-26.00)	0.052
BI score, median (IQR)	80.00 (50.00-90.00)	80.00 (60.00-95.00)	70.00 (40.00-90.00)	0.068
mRS score, median (IQR)	2.00 (2.00-3.00)	2.00 (2.00-3.00)	3.00 (2.00-4.00)	0.015
HAMD score, median (IQR)	4.00 (2.00-6.00)	5.00 (2.00-6.00)	6.00 (3.00-9.25)	< 0.001

WBC, White blood cell; N, neutrophil; L, lymphocyte; NLR, neutrophil-to-lymphocyte ratio; TG, total triglyceride; TC, total cholesterol; LDL-C, low density lipoprotein cholesterol; HDL-C, high density lipoprotein cholesterol; ApoA, apolipoprotein A; ApoB, apolipoprotein B; Hcy, homocysteine; NHHR, Non-High-Density Lipoprotein Cholesterol to High-Density Lipoprotein Cholesterol Ratio; BI, Barthel Index; mRS, modified Rankin Scale; NIHSS, National Institute of Health Stroke Scale; MMSE, Mini-Mental State Examination; IQR, inter-quartile range.

**Table 3:** NHHR tertiles of patients.

Variables	Depression (n=93)	Non-depression (n=315)	χ2	*P* value
NHHR				<0.001
Tertile1 (1.18-7.68)	10 (10.75%)	126 (40.00%)	27.639	<0.001
Tertile2 (7.69-10.50)	24 (25.81%)	112 (35.56%)	3.071	0.080
Tertile3 (10.52-31.80)	59 (63.44%)	77 (24.44%)	49.135	<0.001

NHHR, Non-High-Density Lipoprotein Cholesterol to High-Density Lipoprotein Cholesterol Ratio.

### 3.3 Association between the level of NHHR and PSD

In **Table 4**, with all patients taken as a whole, PSD occurrence taken as a dependent variable and lowest tertile taken as the reference was used for NHHR in the unadjusted and multivariate-adjusted logistic regression models. In the unadjusted model, higher NHHR levels were significantly associated with an increased risk of PSD. Participants in the highest tertile (Q3) exhibited a nearly tenfold increase in depression risk compared to the reference group (OR 9.655, 95% CI 4.663–19.989, *P* < 0.001). This robust association persisted across all adjusted models. After controlling for demographic factors (Model 1), the OR for the highest tertile remained highly significant (OR 11.028, 95% CI 5.235–23.229, *P* < 0.001). Further adjustment for vascular risk factors in Model 2 yielded an OR of 11.450 (95% CI 5.405–24.253, *P* < 0.001). Finally, in the fully adjusted model (Model 3), which included stroke severity (NIHSS, mRS) and basal ganglia lesions, the highest NHHR tertile remained a potent independent predictor of PSD (OR 3.240, 95% CI 1.850–5.680, *P* < 0.001).

**Table 4:** Multivariate logistic regression analysis model of NHHR and PSD.

	Tertile	OR^a^	95% CI	*P* value
Unadjusted	Middle	2.700	1.237-5.892	0.013
	Highest	9.655	4.663-19.989	*P*<0.001
Model 1b	Middle	3.293	1.462-7.417	0.004
	Highest	11.028	5.235-23.229	*P*<0.001
Model 2c	Middle	3.243	1.439-7.304	0.012
	Highest	11.450	5.405-24.253	*P*<0.001
Model 3d	Middle	2.619	1.120-4.150	0.018
	Highest	3.240	1.850-5.680	*P*<0.001

NHHR, Non-High-Density Lipoprotein Cholesterol to High-Density Lipoprotein Cholesterol Ratio; OR, odds ratio; CI, confidence level; PSD, post-stroke depression;.

a Reference OR (1.000) is the lowest tertile of NHHR for PSD.

b Model 1: adjusted for age, sex, education years, married.

c Model 2: adjusted for covariates from Model 1 and further adjusted for Vascular risk factors (Hypertension, diabetes mellitus, coronary heart disease, atrial fibrillation, current smoking, alcohol consumption, prior stroke).

d Model 3: adjusted for covariates from Model 2 and further adjusted for

variables with *p* < 0.05 in univariate analysis (basal ganglia lesions, baseline NIHSS score, and mRS score).

### 3.4 ROC curve analysis of admission NHHR for predicting PSD

To quantify the diagnostic predictive performance of admission NHHR for the development of PSD at 3 months post-stroke, we performed a conventional ROC curve analysis (**Figure 2**). The admission NHHR level exhibited a robust predictive capacity for PSD, with an AUC of 0.738 (95% CI: 0.681–0.790, *P* < 0.001). According to the Youden index, the optimal cut-off value of NHHR for predicting PSD was 10.180. At this threshold, the predictive sensitivity was 71.0% and the specificity was 69.5% (**Figure 2**).

**Figure 2:**
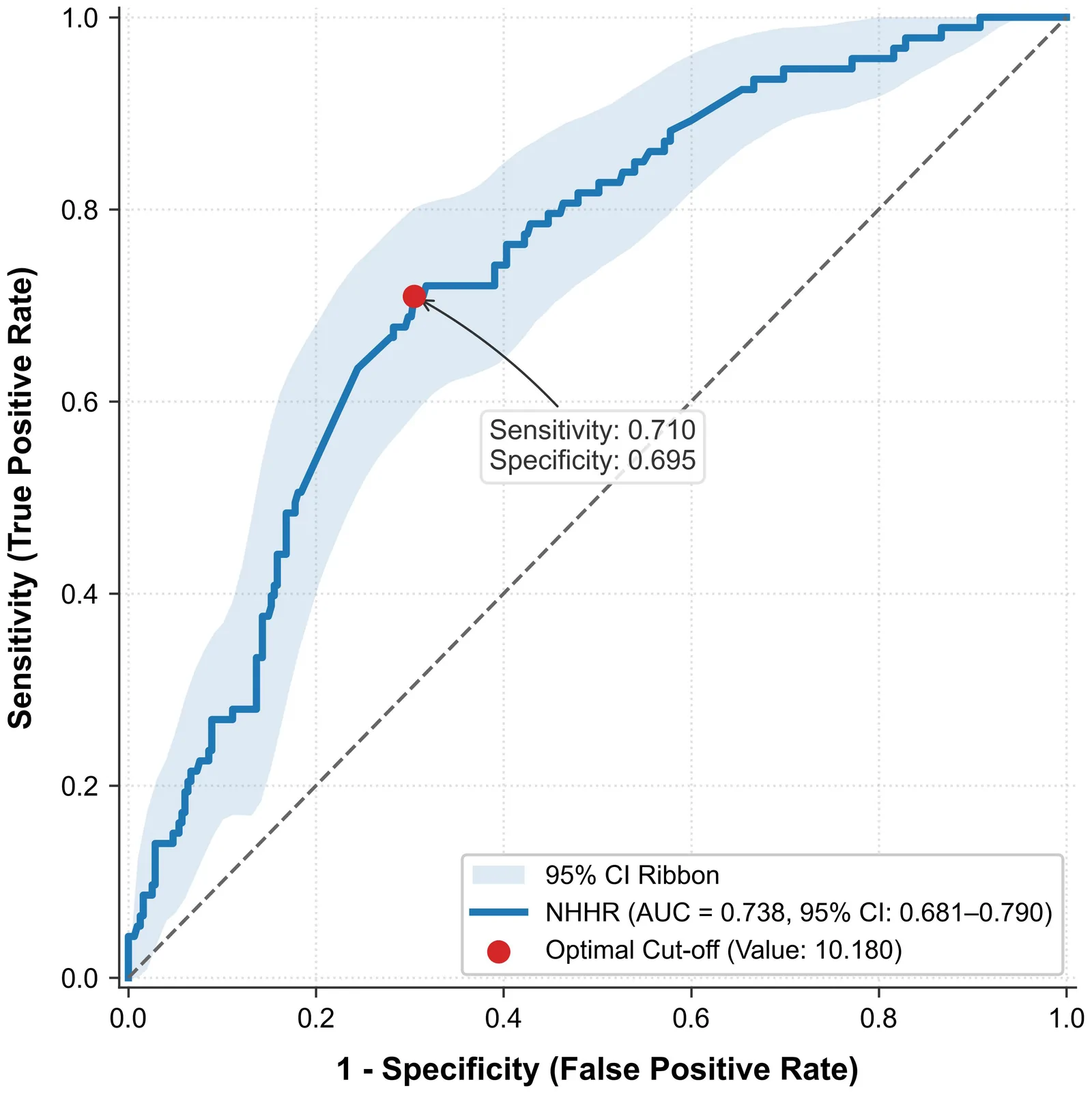
Receiver operating characteristic curves for predicting of depression following AIS.

### 3.5 Detection of nonlinear relationships

To elucidate potential non-linearities in the association between NHHR and 90-day PSD, we employed RCS analysis integrated within a multivariable-adjusted logistic regression framework. Our results revealed a statistically significant non-linear relationship (*P* for overall < 0.001; *P* for non-linearity < 0.001; **Figure 3**). Specifically, the spline architecture demonstrated a monotonically increasing risk of PSD with rising NHHR levels, characterized by a pronounced inflection point. The risk escalation was notably more rapid within the NHHR range of 10 to 15, subsequently transitioning to a more gradual ascent at higher values, where the wider confidence intervals reflected diminished sample density or increased variability in the upper tail.

**Figure 3:**
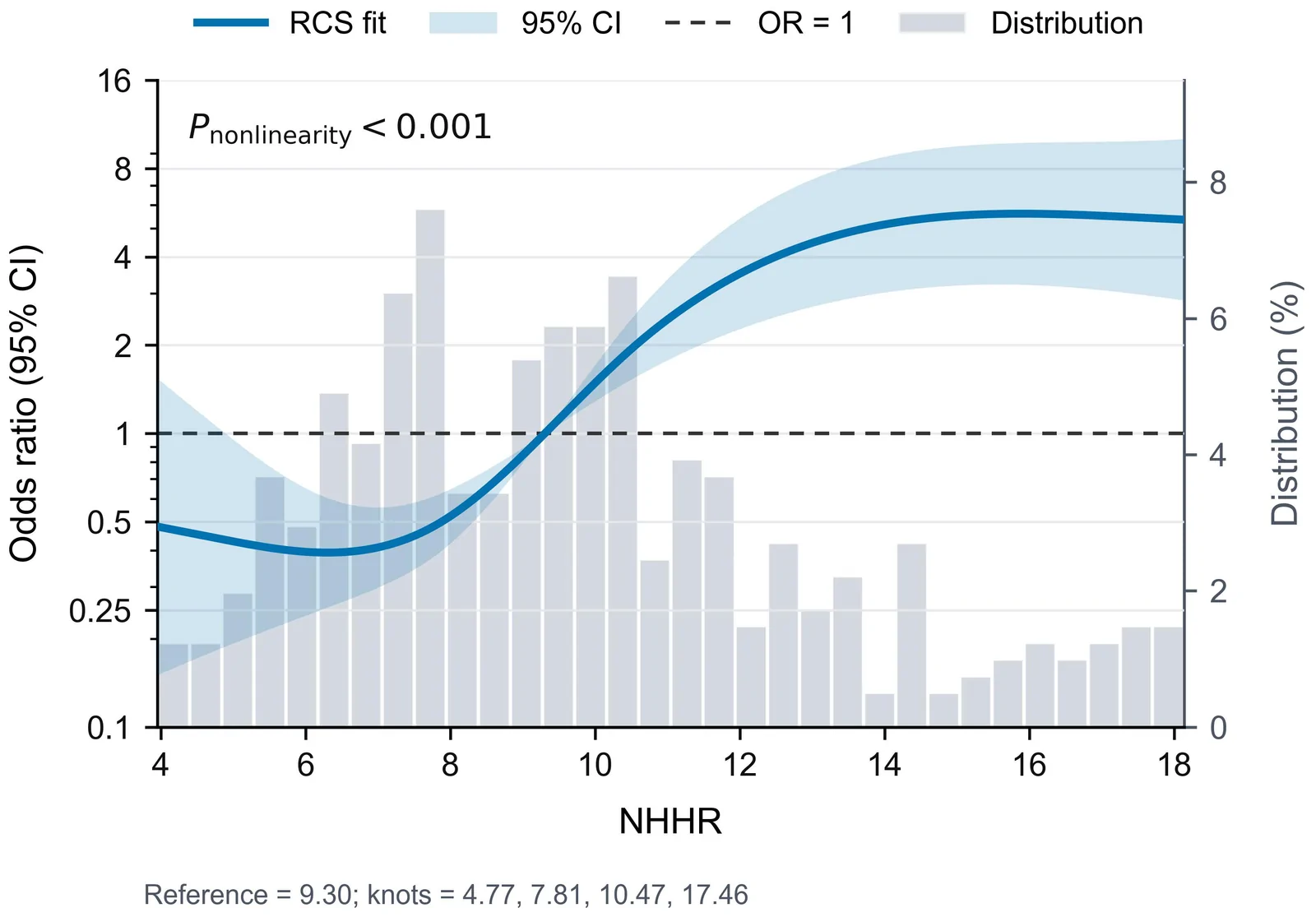
RCS evaluated the nonlinear association of NHHR with 90-day.

### 3.6 Subgroup analyses

To evaluate the stability of the association between admission NHHR and PSD across diverse patient populations, we performed comprehensive subgroup analyses stratified by key demographic and clinical characteristics (**Figure 4**). The positive association between elevated NHHR and increased PSD risk remained robust and statistically significant across several major subgroups, including patients aged ≥ 65 years (OR 1.17, 95% CI 1.04–1.32, *P* = 0.012), patients with basal ganglia lesions (OR 1.25, 95% CI 1.05–1.49, *P* = 0.013), patients with moderate-to-severe stroke (NIHSS > 4) (OR 2.01, 95% CI 1.02–3.10, *P* = 0.014), and those with poor functional outcomes (mRS > 2) (OR 2.03, 95% CI 1.21–2.96, *P* = 0.018). Despite these intra-subgroup variations, formal interaction testing revealed no significant effect modification by any of the stratified clinical variables (all *P* for interaction > 0.05).

**Figure 4:**
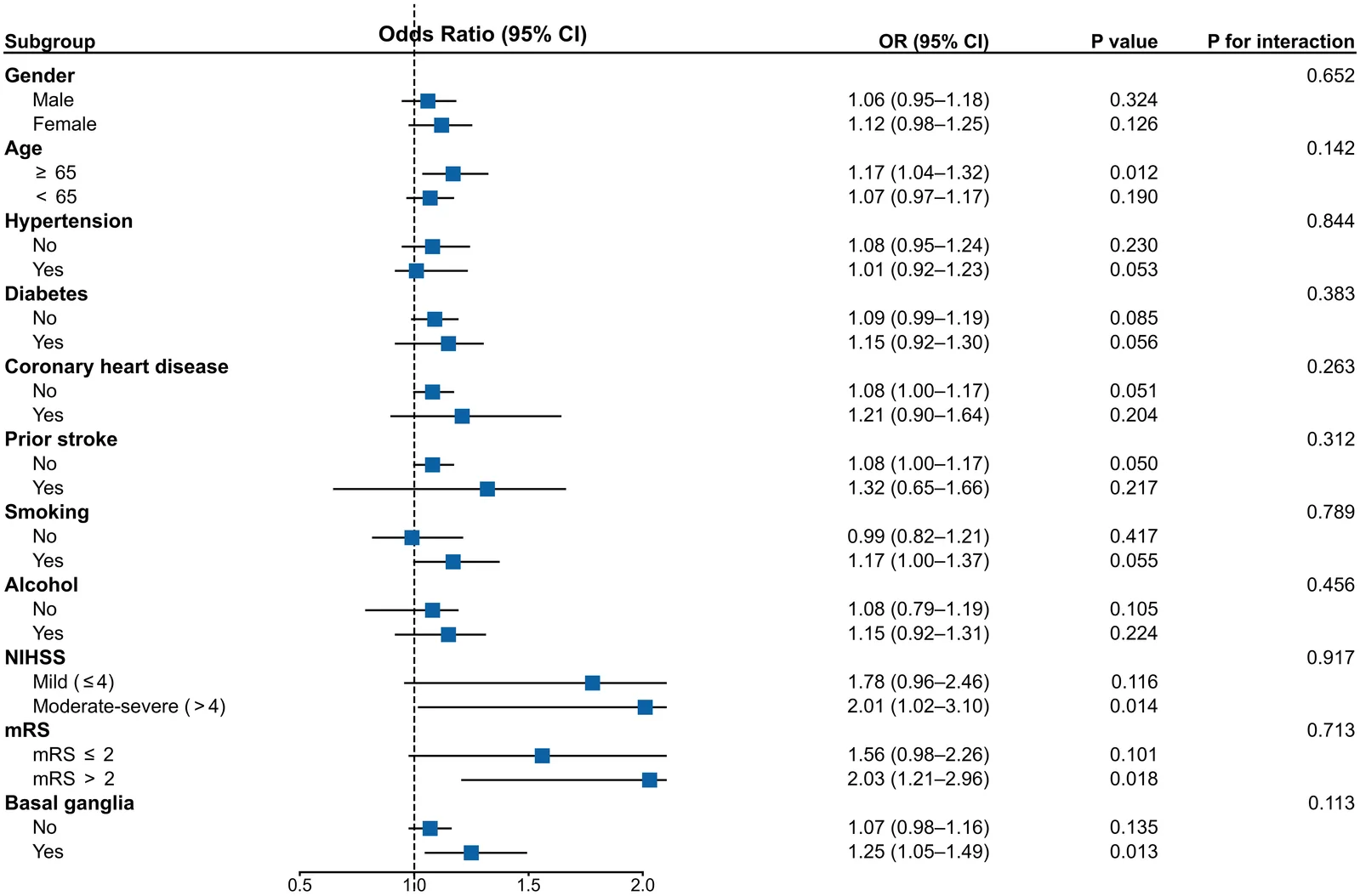
Subgroup analysis of the association between NHHR and PSD.

## 4 Discussion

Our findings demonstrate that patients in the highest tertile of NHHR at admission faced a 3.240-fold higher risk of PSD compared to those in the lowest tertile, even after rigorous adjustment for stroke severity, functional impairment, and baseline inflammatory markers. Notably, RCS analysis revealed a statistically significant non-linear, monotonically increasing relationship, with a pronounced risk escalation observed between NHHR values of 10 and 15. To our knowledge, this investigation represents the first clinical evidence characterizing NHHR—a comprehensive marker of lipid-driven systemic inflammation—as a novel predictor for PSD, suggesting that dysregulated lipid metabolism and immune activation are central to the neuropsychiatric sequelae of cerebrovascular events.

The incidence of PSD in our cohort was 22.79%, closely approximating global meta-analyses which indicate that roughly one-third of stroke survivors manifest depressive sequelae (5, 25). Despite this substantial prevalence, PSD remains frequently underdiagnosed and inadequately managed, ultimately impeding functional recovery, exacerbating cognitive attrition, and escalating post-stroke mortality. While our findings corroborate the established role of stroke severity (NIHSS) and functional disability (mRS) as conventional risk factors (26, 27), they further elucidate the neuroanatomical substrates involved. Specifically, the basal ganglia and frontal regions represent pivotal nodes within the human emotional network; structural disruption to these areas is inherently linked to the emergence of affective symptoms. This is consistent with the seminal work by Chatterjee et al., who delineated a robust correlation between basal ganglia lesions and depressive phenotypes (28). However, the striking predictive efficacy of the NHHR suggests that biochemical vulnerability may be as fundamental as macrostructural damage in the ontogeny of post-ischemic mood disorders. The NHHR transcends individual lipid parameters by integrating the dynamic equilibrium between pro-atherogenic, pro-inflammatory lipoproteins (non-HDL-C) and anti-inflammatory, vasculoprotective fractions (HDL-C) (29, 30). Unlike traditional markers such as LDL-C, which primarily reflect cholesterol transport, the NHHR provides a holistic signature of the atherogenic burden and the systemic inflammatory milieu. Notably, we observed a progressive escalation of PSD incidence across NHHR tertiles, spanning from 10.75% in the lowest tertile to 63.44% in the highest.

Our comprehensive subgroup analyses provide novel insights into the population heterogeneity of the NHHR-PSD association. While interaction tests indicated no fundamental shift in the overall predictive direction of NHHR (all *P* for interaction > 0.05), we observed that the association was particularly pronounced in specific high-risk subsets, notably older patients (≥ 65 years), those with basal ganglia lesions, and individuals with greater neurological (NIHSS > 4) or functional (mRS > 2) impairments. The heightened sensitivity of younger patients to lipid-driven systemic inflammation may reflect distinct age-related neuro-immune responses or different baseline metabolic reserves. Similarly, the basal ganglia are integral components of the cortico-striato-pallido-thalamic circuits governing mood and reward; thus, ischemic damage to this region, compounded by the intense systemic inflammatory milieu indicated by elevated NHHR, may synergistically precipitate depressive phenotypes. Conversely, the attenuated predictive value in younger patients or those with mild strokes suggests that in these groups, PSD pathogenesis might be more heavily influenced by cumulative age-related neurodegeneration or pre-existing microvascular burdens rather than acute lipid-inflammatory fluctuations. These nuanced findings underscore the necessity of interpreting NHHR within the broader clinical context and advocate for personalized, biomarker-guided psychiatric surveillance tailored to the patient’s specific demographic and neuroanatomical profile.

The robust association between NHHR and PSD may be related to the lipid inflammation-neurodegeneration triad. While our study did not directly measure inflammatory cytokines (e.g., IL-6, CRP, TNF-α), existing literature proposes that high levels of proatherogenic lipids (reflected in high NHHR) can drive systemic low-grade inflammation, which in turn may exacerbate neuroinflammation and affect mood regulation circuits (31–33). We emphasize that these mechanistic interpretations remain speculative and warrant direct empirical investigation in future studies. NHHR is a comprehensive reflection of the balance between pro-atherogenic and anti-atherogenic lipoproteins. Non-HDL-C encompasses all potentially atherogenic particles, including LDL, VLDL, and intermediate-density lipoproteins. High levels of these lipids are not merely vascular risks; they are drivers of systemic low-grade inflammation. In AIS, the sudden vascular occlusion triggers a cascade of oxidative stress and the release of damage-associated molecular patterns (34, 35). Elevated Non-HDL-C levels exacerbate this by promoting monocyte activation and the subsequent secretion of pro-inflammatory cytokines such as interleukin-1β, IL-6, and tumor necrosis factor-α (36). These cytokines are known to cross the blood-brain barrier or signal through the vagus nerve to modulate neurocircuitry associated with mood regulation, such as the hypothalamic-pituitary-adrenal axis (37). Conversely, HDL-C is recognized for its potent anti-inflammatory and antioxidant properties (38). HDL-C facilitates cholesterol efflux, inhibits the expression of adhesion molecules on endothelial cells, and suppresses the activation of CD11b on monocytes (39). Our findings showed significantly lower HDL-C levels in PSD patients, suggesting a failure in these protective mechanisms. When the ratio is high (high NHHR), the systemic environment shifts toward a pro-inflammatory state. Oxidative stress can trigger lipid and protein peroxidation, compromising the synthesis and metabolism of neurotransmitters and ultimately leading to pathological alterations within the nervous system. Notably, a deficiency in anti-inflammatory and antioxidant defenses—resulting from diminished HDL-C levels—may render the brain more vulnerable to these deleterious mechanisms. Consequently, dyslipidemia, particularly the imbalance between HDL-C and LDL-C, may increase the risk of PSD through pathways involving systemic inflammation and oxidative stress.

A critical highlight of our study is the RCS analysis, which unveiled a statistically significant non-linear relationship between NHHR and PSD risk. We identified a “vulnerability window” between NHHR values of 10 and 15, where the risk of depression escalates most sharply—suggesting a critical threshold of lipid-driven inflammation beyond which the brain’s compensatory mechanisms for emotional regulation are overwhelmed. The transition from a rapid ascent to a more gradual increase at higher NHHR levels may reflect a saturation effect of inflammatory receptors or a ceiling effect in the clinical manifestation of depressive symptoms. For clinicians, this inflection point serves as a potential “red flag” for early psychiatric intervention. The incremental novelty of our work lies in the integration of RCS and ROC curve analyses. Unlike previous studies that merely established linear risk correlations, we not only identified this clear non-linear relationship with a sharp risk escalation window (NHHR: 10–15) but also validated a precise clinical threshold of 10.180 (AUC = 0.738, sensitivity = 71.0%, specificity = 69.5%). This dual quantification provides clinicians with a highly accessible, low-cost “red flag” at admission, facilitating early psychiatric screening and patient-tailored preventive care.

While individual markers like LDL-C or HDL-C have yielded inconsistent results in psychiatric literature, the NHHR integrates the total atherogenic lipid burden. Previous studies have noted that higher levels of Non-HDL-C are associated with generalized anxiety disorder and major depressive disorder. Our study extends this to the stroke population, showing that the NHHR is a more sensitive prognostic tool than its individual components. By adjusting for NIHSS and mRS in Model 3, we demonstrated that the predictive power of NHHR is not merely a reflection of larger infarcts, but rather a reflection of the systemic metabolic milieu that shapes the brain’s psychological recovery. A study involving 373 patients with AIS, which assessed serum lipid profiles within 24 hours of admission, demonstrated that decreased HDL-C levels were associated with an elevated risk of PSD—a finding directly congruent with our proposed hypothesis (40). Furthermore, a cross-sectional study of 411 stroke patients revealed that a higher monocyte-to-HDL-C ratio was significantly correlated with increased PSD risk, thereby providing indirect evidence for the link between low HDL-C levels and PSD development (41). In contrast, a notable epidemiological study derived from the 2010–2012 Korea National Health and Nutrition Examination Survey (KNHANES) reported that elevated HDL-C levels were significantly associated with an increased prevalence of depressive symptoms (42). Because higher HDL-C levels naturally lead to a lower calculated NHHR, the KNHANES conclusion contradicts our current observation that low HDL-C (yielding higher NHHR) drives depression. This contradiction can be systematically unpackaged by evaluating the distinct epidemiological contexts and underlying pathophysiological profiles of the two cohorts. The KNHANES database examined a stable, community-dwelling general population where elevated HDL-C often reflects chronic lifestyle behaviors (e.g., alcohol consumption), lipid-altering medications, or the accumulation of dysfunctional, large, spherical, pro-inflammatory HDL sub-fractions common in chronic metabolic syndromes. In contrast, our cohort evaluates patients in the acute phase of an ischemic stroke emergency. In an AIS setting, circulating functional HDL-C is rapidly depleted as it is consumed to counteract massive systemic oxidative stress and stabilize broken cerebral endothelial barriers. Consequently, during acute brain ischemia, a low baseline HDL-C level (and therefore a highly elevated NHHR) serves as a direct proxy for a collapsed peripheral anti-inflammatory reservoir, leaving the post-stroke brain unprotected against thrombo-inflammatory cascades that cause affective neural network failure.

There were several limitations on interpretation of its results. First, lipid profiles and NHHR were measured only within 24 hours of admission. Chronic inflammation is dynamic; future studies should employ serial measurements to track the NHHR trajectory during the 3-month recovery period to determine if persistent elevation carries a worse prognosis. Second, we excluded patients with severe aphasia or dysarthria to ensure the validity of the HAMD scores. Given that these patients often have the most severe strokes and potentially the highest inflammatory burdens, our results may actually underestimate the true incidence and risk associated with NHHR. Third, While we adjusted for major clinical variables, we did not routinely screen for specific inflammatory cytokines (e.g., IL-6, C-reactive protein) or genetic polymorphisms (e.g., APOE4) that could further elucidate the mechanism.

Fourth, the exploratory and single-center nature of this prospective observational cohort means that the protocol was not pre-registered in a public clinical trial registry at its inception, which represents an administrative limitation in our early standardization workflow. Finally, the 3-month window is standard for PSD diagnosis, but early-onset depression (within 1 month) might be driven by different biological stressors than late-onset depression.

## 5 Conclusions

In conclusion, elevated admission NHHR is independently and non-linearly associated with an increased risk of PSD at three months in AIS patients. As an easily accessible biomarker of lipid-driven systemic inflammation, NHHR may serve as a valuable prognostic tool for early risk stratification and targeted psychological intervention in clinical practice.

## Data Availability

The original contributions presented in the study are included in the article/supplementary material, further inquiries can be directed to the corresponding author/s.
